# Oxidative Stress: A Unifying Paradigm in Hypertension

**DOI:** 10.1016/j.cjca.2020.02.081

**Published:** 2020-05

**Authors:** Rhian M. Touyz, Francisco J. Rios, Rhéure Alves-Lopes, Karla B. Neves, Livia L. Camargo, Augusto C. Montezano

**Affiliations:** Institute of Cardiovascular and Medical Sciences, British Heart Foundation Glasgow Cardiovascular Research Centre, University of Glasgow, Glasgow, Scotland, United Kingdom

## Abstract

The etiology of hypertension involves complex interactions among genetic, environmental, and pathophysiologic factors that influence many regulatory systems. Hypertension is characteristically associated with vascular dysfunction, cardiovascular remodelling, renal dysfunction, and stimulation of the sympathetic nervous system. Emerging evidence indicates that the immune system is also important and that activated immune cells migrate and accumulate in tissues promoting inflammation, fibrosis, and target-organ damage. Common to these processes is oxidative stress, defined as an imbalance between oxidants and antioxidants in favour of the oxidants that leads to a disruption of oxidation-reduction (redox) signalling and control and molecular damage. Physiologically, reactive oxygen species (ROS) act as signalling molecules and influence cell function through highly regulated redox-sensitive signal transduction. In hypertension, oxidative stress promotes posttranslational modification (oxidation and phosphorylation) of proteins and aberrant signalling with consequent cell and tissue damage. Many enzymatic systems generate ROS, but NADPH oxidases (Nox) are the major sources in cells of the heart, vessels, kidneys, and immune system. Expression and activity of Nox are increased in hypertension and are the major systems responsible for oxidative stress in cardiovascular disease. Here we provide a unifying concept where oxidative stress is a common mediator underlying pathophysiologic processes in hypertension. We focus on some novel concepts whereby ROS influence vascular function, aldosterone/mineralocorticoid actions, and immunoinflammation, all important processes contributing to the development of hypertension.

## Résumé

L'étiologie de l'hypertension implique des interactions complexes entre les facteurs génétiques, environnementaux et physiopathologiques qui influencent de nombreux systèmes de régulation. L'hypertension est typiquement associée à une dysfonction vasculaire, à un remodelage cardiovasculaire, à une dysfonction rénale et à une stimulation du système nerveux sympathique. De nouvelles données indiquent que le système immunitaire est également important et que les cellules immunitaires activées migrent et s'accumulent dans les tissus, favorisant l'inflammation, la fibrose et la lésion des organes cibles. Ces processus ont en commun le stress oxydatif, défini comme étant un déséquilibre entre les oxydants et les antioxydants en faveur des oxydants qui conduit à une perturbation de la signalisation et du contrôle de l'oxydoréduction (redox) et à des dommages moléculaires. Physiologiquement, les espèces réactives de l'oxygène (ERO) agissent comme des molécules de signalisation et influencent la fonction cellulaire par une transduction du signal hautement régulée et sensible à l'oxydoréduction. Dans l'hypertension, le stress oxydatif favorise la modification post-traductionnelle (oxydation et phosphorylation) des protéines et une signalisation aberrante avec des dommages conséquents aux cellules et aux tissus. De nombreux systèmes enzymatiques génèrent des ERO, mais les NADPH oxydases (Nox) en sont les principales sources dans les cellules du cœur, des vaisseaux, des reins et du système immunitaire. L'expression et l'activité des Nox sont accrues en cas d'hypertension et sont les principaux systèmes responsables du stress oxydatif dans les maladies cardiovasculaires. Nous présentons ici un concept unificateur dans lequel le stress oxydatif est un médiateur commun qui sous-tend les processus physiopathologiques de l'hypertension. Nous nous concentrons sur quelques nouveaux concepts selon lesquels les ERO influencent la fonction vasculaire, les actions de l'aldostérone et des minéralocorticoïdes, et l'immuno-inflammation, autant de processus importants contribuant au développement de l'hypertension.

Hypertension is a complex, multifactorial, and multisystem disorder as originally described by Irvine Paige in his mosaic theory when he proposed that high blood pressure involves interplay among many elements, including genetic, environmental, anatomic, adaptive, neural, endocrine, humoral, and hemodynamic factors.^1^ Since then, there has been enormous progress in discovering the molecular and cellular processes that connect the numerous components underlying hypertension. In 2013, David Harrison revisited Paige’s mosaic theory, highlighting common molecular mechanisms, specifically oxidative stress and inflammation, as major drivers coordinating diverse cellular events and organ systems in hypertension.^2^

Oxidative stress is characterized by excessive production of reactive oxygen species (ROS) and altered oxidation-reduction (redox) state. These molecular events induce protein oxidation and dysregulated cell signalling, leading to inflammation, proliferation, apoptosis, migration, and fibrosis, which are important processes contributing to impaired vascular function, cardiovascular remodelling, renal dysfunction, immune cell activation, and sympathetic nervous system excitation in hypertension.^1^, ^2^, ^3^, ^4^ A major source of cardiovascular ROS is a family of nonphagocytic NADPH oxidases (Nox1, Nox2, and Nox4 in rodents and Nox1, Nox2, Nox4, and Nox5 in humans).^5^^,^^6^ Expression and activation of Nox isoforms are increased in hypertension and are a likely cause of oxidative stress in cardiovascular, renal, and immune cells in hypertension-associated target organ damage.^6^, ^7^, ^8^ Other enzymatic sources of ROS include mitochondrial oxidases, xanthine oxidase, endoplasmic reticular oxidases, and uncoupled nitric oxide synthase (NOS).

**Figure 1:**
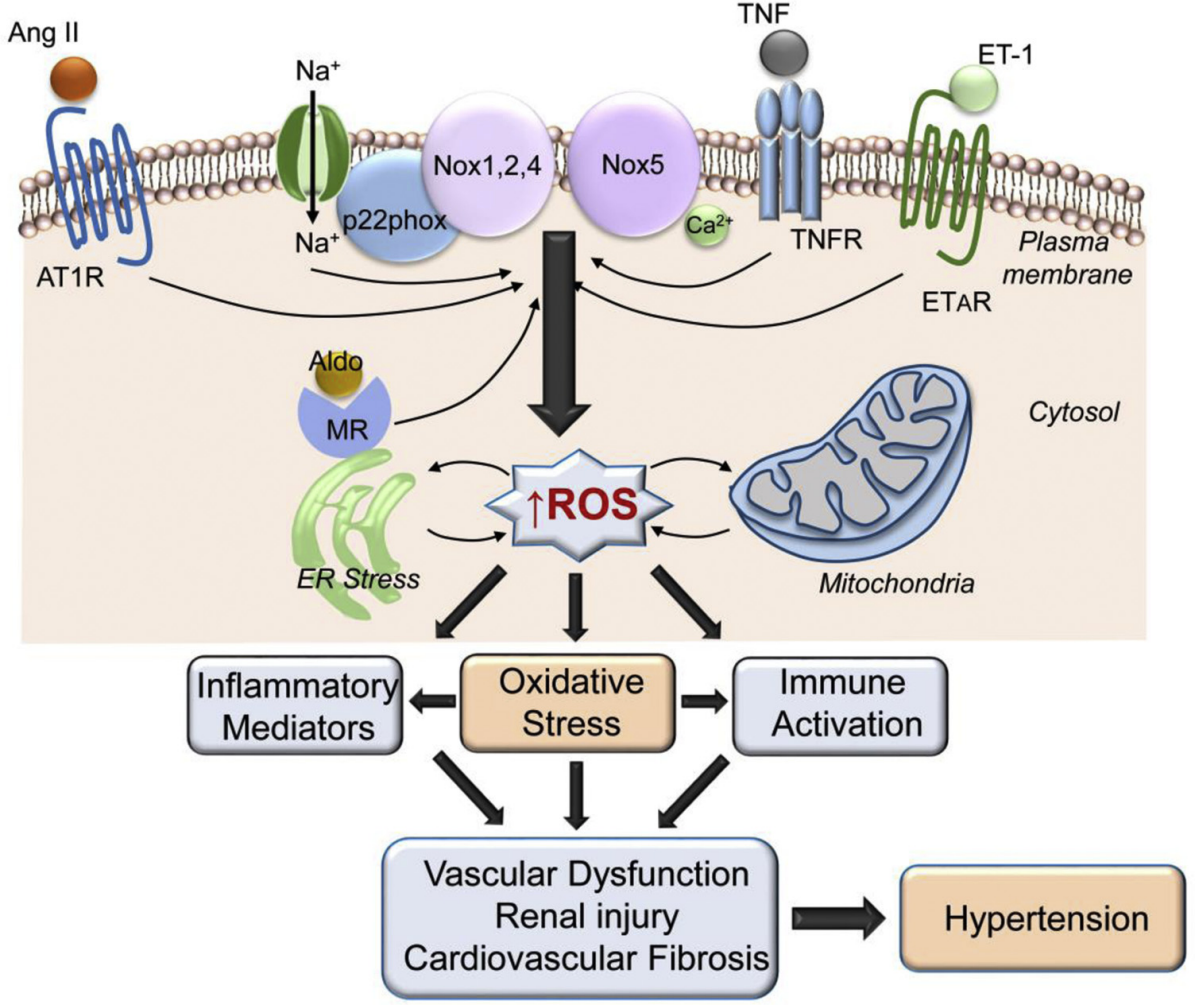
Oxidative stress as a unifying factor in hypertension. Prohypertensive factors, eg, angiotensin II (Ang II), endothelin-1 (ET-1), aldosterone (Aldo), and salt (Na), induce activation of NADPH oxidases (Noxs) that generate reactive oxygen species (ROS), which influence multiple systems involved in the pathophysiology of hypertension. AT1R, angiotensin II type 1 receptor, ER, endoplasmic reticulum, ETAR, endothelin-1 type A receptor; MR, mineralocorticoid receptor; TNF, tumour necrosis factor; TNFR, tumour necrosis factor receptor.

Whereas the ROS-generating role of non-NADPH oxidases in cardiovascular cells seems to be minor in physiologic conditions,^9^ growing evidence suggests that ROS generated in mitochondria and the endoplasmic reticulum (ER) may contribute to oxidative stress in hypertension.^10^, ^11^, ^12^ This likely involves cross-talk between Noxs and mitochondria/ER. In particular, the concept of ROS-induced ROS release (RIRR) may be important, whereby ROS formed in one region activate ROS in another region^13^ (Fig. 1). A number of pharmacologic strategies have been developed to lower cross-talk between Noxs and mitochondria, which may reduce RIRR.^14^ Mitochondrial oxidative stress–induced endothelial dysfunction in hypertension has been attributed to reduced sirtuin 3 (SIRT3)–mediated superoxide dismutase 2 (SOD2) signalling.^15^ These processes were ameliorated by restoration of SIRT3, suggesting that SIRT3 influences the mitochondrial redox state by regulating mitochondrial antioxidant systems. Mito-Tempol, an SOD mimetic, accumulates in mitochondria and has been shown to improve endothelial function, inhibit ROS production, and reduce blood pressure in experimental hypertension.^10^ Moreover, recent studies in humans showed that chronic supplementation with a mitochondrial antioxidant (MitoQ) improves vascular function in aged individuals.^16^ Mitochondrial dysfunction and ROS generation in the brain seem to be especially important in neurogenic hypertension, where neural mitochondrial biogenesis and bioenergetics influence sympathetic outflow to the cardiovascular system.^17^

The ER has also been implicated in oxidative stress in hypertension. We demonstrated that vascular hypercontractility in stroke-prone spontaneously hypertensive rats (SHR-SPs) involves oxidative and ER stress through Nox4-dependent processes.^18^ Inhibition of ER stress with the use of 4-phenylbutyric acid (4-PBA) and STF083010 (an IRE1-XBP1 disruptor) ameliorated vascular dysfunction in SHR-SPs.^18^ Treatment of SHRs with 4-PBA reduced blood pressure and improved vascular function and structure by ameliorating ER stress.^12^ Although ER- and mitochondria-derived ROS may contribute in part to oxidative stress in hypertension, the upstream driving factor appears to be Nox activation.^13^^,^^14^

Oxidative stress and altered redox signalling are emerging as major pathogenic factors in cardiovascular disease. This review examines the role of cellular oxidants in the cardiovascular system and focuses on oxidative stress as a common molecular process in some pathophysiologic events underlying hypertension. In particular, we discuss some novel concepts related to the central role of ROS in vascular function, hyperaldosteronism, and inflammation in hypertension. ROS also influence many other systems involved in hypertension, and the reader is referred to recent papers for further details.^2^, ^3^, ^4^, ^5^, ^6^, ^7^, ^8^

## Reactive Oxygen Species, Oxidative Stress, and Redox Signalling in Hypertension

In physiologic conditions, ROS are intimately involved in and required for normal biological function, in large part through tightly controlled redox regulation, redox signaling and redox sensing.^19^ In pathological conditions, uncontrolled ROS production leads to oxidative stress defined as “an imbalance between oxidants and antioxidants in favor of the oxidants, leading to a disruption of redox signaling and control and/or molecular damage.”^20^ In the cardiovascular system, the most important ROS are superoxide anion (O_2_^−^), hydrogen peroxide (H_2_O_2_), nitric oxide (NO), and peroxynitrite (ONOO^−^).^19^, ^20^, ^21^ Although O_2_^−^ is highly unstable and cell membrane impermeable, H_2_O_2_ is cell membrane permeable, is stable, and has a longer half-life than O_2,_^−^ making it an efficient signalling molecule.^22^^,^^23^ NO, produced enzymatically by NOS, is the prototype endothelial-derived vasodilator.^24^^,^^25^ When NO reacts with O_2,_^−^ it forms ONOO,^−^ a strong oxidant that is highly unstable.^26^ When protonated (HOONO), peoxynitrite is cell membrane permeable. The interplay between O_2_^−^ and NO, together with dysregulated production of O_2_^−^ and H_2_O_2_, contributes to altered cellular redox status and oxidative damage of cells and tissues.^27^

ROS influence cell function by modifying proteins through posttranslational modifications, such as oxidation (sulfenylation, nitrosylation, glutathionylation, and carbomylation) and phosphorylation.^28^, ^29^, ^30^ Proteins that are redox sensitive include ion transporters, receptors, signalling molecules, transcription factors, cytoskeletal structural proteins, and matrix metalloproteases, all of which are involved in regulating vascular, cardiac, and renal functions.^30^^,^^31^ ROS are key signalling molecules through which vasoactive agents such as angiotensin II (Ang II), endothelin-1 (ET-1), aldosterone, and prostanoids mediate cellular effects, and they regulate intracellular calcium homeostasis,^32^, ^33^, ^34^, ^35^ which is important in triggering and maintaining vasoconstriction and cardiac contraction. ROS activate all 3 members of the mitogen-activated protein kinase (MAPK) family, including ERK1/2, p38MAPK, and JNK, which control cardiac and vascular cell proliferation, migration, hypertrophy, and inflammation.^34^, ^35^, ^36^, ^37^ ROS also influence tyrosine kinases important in cardiovascular inflammation and fibrosis, such as c-Src, PI3K/Akt, FAK, and receptor tyrosine kinases (VEGFR, EGFR, PDGFR), and they are critically involved in oxidation of actin and actin-associated proteins important in cystoskeletal organization.^38^, ^39^, ^40^ ROS stimulate activation of transcription factors (nuclear factor [NF] κB, signal transducer and activator of transcription [STAT] activator protein 1 [AP-1], and hypoxia-inducible factor 1 [HIF-1]), proinflammatory genes, chemokine and cytokine production, and recruitment and activation of inflammatory and immune cells that promote cardiovascular and renal inflammation and fibrosis,^41^, ^42^, ^43^ which are important processes underlying vascular injury and target-organ damage in hypertension. Protein phosphatases (protein tyrosine phosphatases and protein serine/threonine phosphatases), which catalyze dephosphorylation of signalling molecules, are highly redox sensitive.^43^ In the oxidised state, protein phosphatases are usually inactive, leading to decreased dephosphorylation of downstream protein targets with consequent increased phosphorylation and activation, processes that are altered in hypertension.^44^

Hydrogen peroxide is an important diffusible second messenger that plays a functional role in intercellular signalling through gap junctions, connexins, and myoendothelial feedback.^45^^,^^46^ H_2_O_2_ mediates effects primarily through oxidation of cysteine thiols. In the endothelium, H_2_O_2_ acts as a vasodilator and has been considered to be an endothelium-derived relaxing factor, effects that are induced largely through activation of PKG1α and posttranslational modifications involving S-glutathionylation.^47^^,^^48^ Increased intracellular H_2_O_2_ promotes phosphorylation of p66Shc, a key mitochondrial ROS regulator that involves vascular endothelial growth factor, which is important in endothelial cell migration, proliferation, and angiogenesis.^49^ In vascular smooth muscle cells, we demonstrated that H_2_O_2_ induces posttranslational modification of ERK1/2 and p38MAPK through tyrosine kinase–dependent, protein kinase C (PKC)–independent mechanisms, processes that are up-regulated in hypertension.^38^ Critical to these events is oxidation of protein tyrosine phosphatases.^50^ H_2_O_2_ has also been implicated in oxidation of cofilin, an actin-associated protein important in cell motility migration and vascular remodelling in hypertension.^51^

## Evidence Supporting a Role for ROS and Oxidative Stress in Hypertension

Oxidative stress has been causally linked to increased blood pressure in various experimental models of hypertension, including genetic hypertension (spontaneously hypertensive rats [SHRs], stroke-prone SHRs [SHR-SPs]), endocrine-induced hypertension [Ang II, aldosterone, deoxycorticosterone acetate (DOCA)]), surgically induced hypertension (2-kidney 1-clip [2K1C], aortic banding), diet-induced hypertension (salt, fat, zinc), neurogenic hypertension, pulmonary hypertension, and preeclampsia.^17^^,^^52^, ^53^, ^54^, ^55^, ^56^ Biomarkers of oxidative stress, including plasma and urinary thiobarbituric acid–reactive substances (TBARS) and F_2α_-isoprostanes, tissue concentrations of O_2_^−^ and H_2_O_2_, and activation of Noxs and xanthine oxidase, are increased, whereas levels of NO and antioxidant enzymes are reduced in experimental hypertension.^52^, ^53^, ^54^, ^55^, ^56^, ^57^ Further supporting a role for oxidative stress in the pathophysiology of hypertension are studies demonstrating blood pressure–lowering effects of antioxidants, ROS scavengers, and Nox inhibitors. Treatment with antioxidant vitamins (vitamins C and E), SOD mimetics (tempol [4-hydroxy-2,2,6,6-tetramethyl piperidinoxyl]), free radical scavengers (*N*-acetyl-l-cysteine), tetrahydrobiopterin, nonspecific Nox inhibitors (apocynin, diphenylene iodinium), and specific Nox inhibitors (gp91dstat, GKT compounds) reduce oxidative stress and ameliorate or prevent development of hypertension and associated target-organ damage.^58^, ^59^, ^60^, ^61^

Although experimental data support an etiologic role for oxidative stress in the development of hypertension, there is still no confirmation that oxidative stress is a primary cause of hypertension in humans. Reasons for this are complex, as we have previously highlighted,^62^ and relate in large part to 1) lack of sensitive and specific assays that can accurately assess redox state in the clinical setting, 2) paucity of mechanistic studies in disease-appropriate human tissue, and 3) absence of pharmacologic agents that specifically target Nox isoforms or ROS that could be used in patients. Nevertheless, there is clear evidence that ROS production is increased in patients with essential hypertension, renovascular hypertension, malignant hypertension, salt-sensitive hypertension, cyclosporine-induced hypertension, and preeclampsia.^63^, ^64^, ^65^ Population-based observational studies reported an inverse relationship between plasma antioxidants and blood pressure, and clinical studies have shown that systolic and diastolic blood pressures correlate positively with biomarkers of oxidative stress (plasma TBARS and 8-epi-isoprostanes) and negatively with antioxidant levels in patients with hypertension.^66^^,^^67^ Plasma levels of asymmetric dimethylarginine (endothelial NOS inhibitor) and the lipid peroxidation product of linoleic acid, 13-hydroxyoctadecadienoic acid, a marker of ROS production, were inversely correlated with microvascular endothelial dysfunction and elevated blood pressure in hypertensive patients.^68^ Endothelial dysfunction, a hallmark of vascular injury in hypertension, is associated with increased vascular ROS production, oxidative stress, and vascular inflammation in patients with hypertension.^69^ ROS production in vascular smooth muscle cells (VSMCs) from arteries of hypertensive patients demonstrated increased Nox activity, higher levels of O_2_^−^ and H_2_O_2_, enhanced Ang II–stimulated redox signalling, and inflammatory responses compared with cells from normotensive counterparts.^70^ There is also evidence that disruption in ER function (ER stress) contributes to oxidative stress through increased O_2_^−^ generation, decreased antioxidants and activation of mitochondrial oxidases.^71^^,^^72^

Causes of oxidative stress in human hypertension are unclear but may relate, in part, to decreased antioxidant capacity and genetic factors.^73^^,^^74^ Correlation between polymorphisms in glutathione-S-transferase (an intracellular antioxidant enzyme) and the risk of essential hypertension has been reported.^74^ Polymorphisms have also been shown in Nox subunits in hypertensive patients.^75^ Individuals with p22phox polymorphisms exhibit altered Nox activity and increased ROS production in human cardiovascular disease.^76^ More recently, genome-wide association study data from more than 450,000 individuals identified Nox4 and Nox5 as novel blood pressure–related genes.^77^ Although oxidative stress is likely not the sole cause of hypertension, it amplifies blood pressure elevation in the presence of other prohypertensive factors, such as Ang II, ET-1, aldosterone, and salt.

## Oxidative Stress, Sex, and Hypertension

It is well known that premenopausal women are protected from hypertension relative to age-matched men and that this protection is lost with menopause.^78^ Although the biological basis for these sex-related differences in hypertension remain unclear, sex hormones, Y chromosome, Ang II, aldosterone, and sex hormone–related signalling play a critical role.^79^, ^80^, ^81^ In addition, growing evidence suggests that oxidative stress may be important in the sexual dimorphism in hypertension.^82^ Both clinical and preclinical studies have demonstrated that biomarkers of oxidative stress are higher in men than in women.^82^, ^83^, ^84^ In nonhuman male animals, blood pressure decreases in response to antioxidants such as tempol and apocynin, whereas female animals are nonresponsive.^82^^,^^84^ Oxidative stress is involved in the development and maintenance of hypertension in male rats but it seems to be important only in the initial development of hypertension in female rats.^82^ In Ang II–induced hypertension in mice, plasma levels of TBARS were increased in male but not in female mice.^84^ Moreover, Ang II induced a significant increase in O_2_^−^ and H_2_O_2_ production in isolated arteries from male but not female mice.^84^ These differences have been attributed to increased activation of Noxs in males and increased antioxidant capacity in females.^85^^,^^86^ It has also been shown that estradiol reduces expression and activity of Noxs and increases expression of antioxidant enzymes superoxide dismutase and glutathione peroxidase.^87^ Accordingly, the blunted oxidative stress–mediated increase in blood pressure in females may be due to increased activation of antioxidant systems and down-regulation of prooxidant systems.^85^, ^86^, ^87^ Taken together, the current data suggest that oxidative stress may be more important in blood pressure elevation in males than in females.

## Oxidative Stress: A Unifying Mechanism in the Hypertension Mosaic

Because ROS are key players in regulating cardiovascular function, it is not surprising that abnormal ROS regulation and oxidative stress play an important role in the pathophysiology of hypertension. Moreover, because oxidative stress influences myriad signalling molecules and pathways in multiple cells, tissues, organs, and systems, it represents a common molecular mechanism unifying the multifactorial mosaic (Fig. 1) that underlies hypertension. Here we focus on some new concepts relating to the central role of oxidative stress in the regulation of vascular function by vasoactive agents and growth factors, aldosterone and signalling through mineralocorticoid receptors, and inflammation and the immune system.

## Vasoactive Factors, Oxidative Stress, and the Vasculature

Impaired endothelium-dependent vasorelaxation, increased vasoconstriction, vascular remodelling and inflammation, reduced distensibility, and increased stiffness are characteristic features of small and large arteries in hypertension and constitute the vascular phenotype, or “vasculopathy,” of hypertension.^62^^,^^88^^,^^89^ Some of these vascular changes occur with physiologic aging, but in hypertension and diabetes the processes are accelerated, leading to “premature vascular aging,” a process that is highly redox sensitive.^90^^,^^91^ These phenomena are dynamic and involve functional (contraction-relaxation) and structural changes (remodelling) that occur at different phases during development of hypertension. They are defined by complex interactions between vascular cells (endothelium, VSMCs, adventitial fibroblasts) and circulating elements, including vasoactive agents, (Ang II, ET-1, aldosterone, dopamine, catecholamines, prostanoids), growth factors (epidermal growth factor [EGF], insulin-like growth factor 1 [IGF-1], platelet-derived growth factor [PDGF]), sex hormones, microRNAs, exosomes, and endothelial progenitor cells.^92^ In addition, risk factors such as salt and fine particulate matter (air pollutants) can induce vascular dysfunction and inflammation in hypertension.^93^^,^^94^ Common to many of these processes is oxidative stress and activation of redox-sensitive signalling pathways.^34^^,^^62^^,^^90^

### Ang II and ET-1

**Figure 2:**
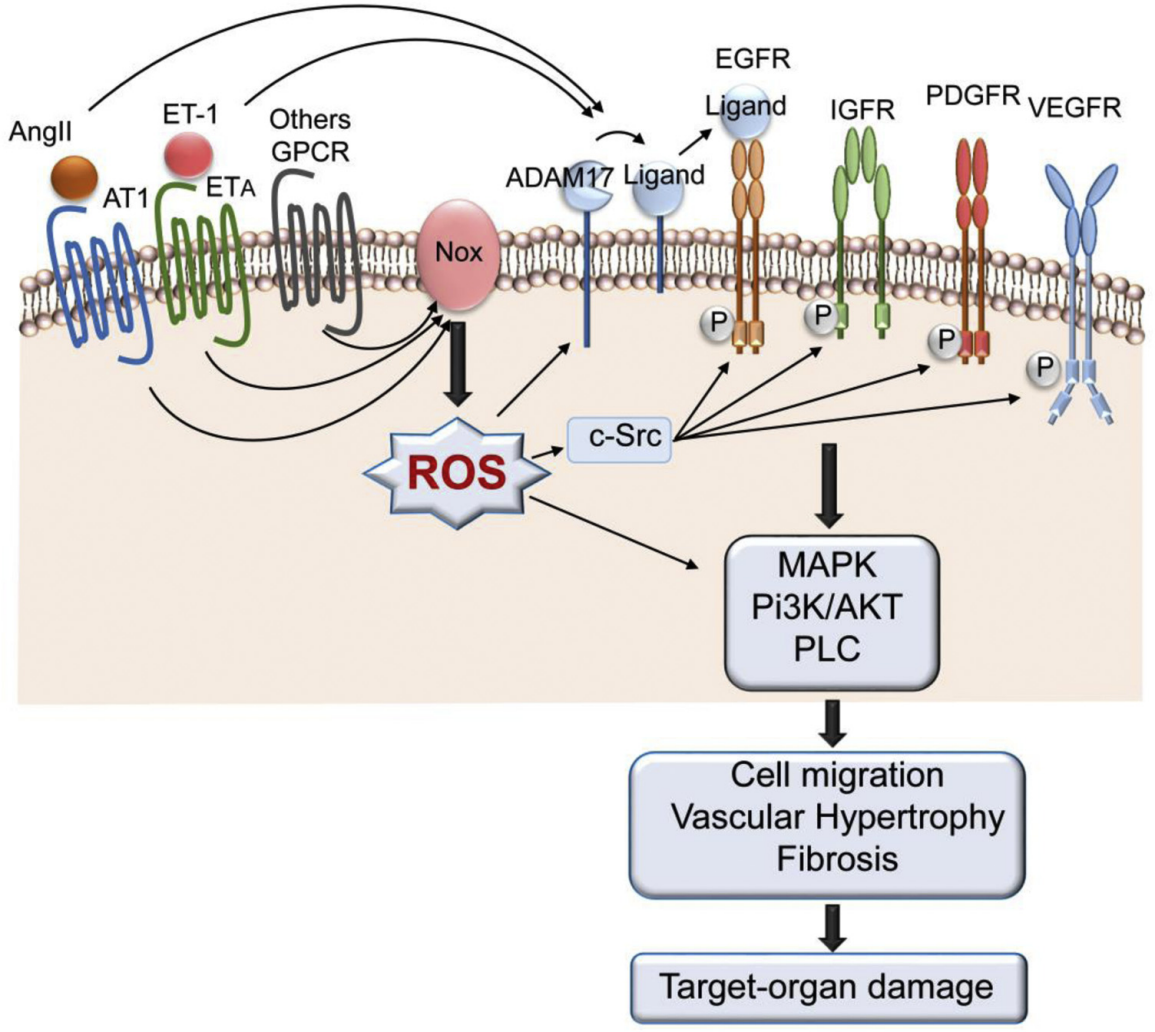
Transactivation of growth factor receptors (GFRs) by angiotensin II (Ang II) and endothelin-1 (ET-1), through their G protein–coupled receptors (GPCRs), stimulate NADPH oxidase (Nox)–derived reactive oxygen species (ROS) production and activation of ROS-signalling pathways that influence cardiovascular processes leading to hypertension-associated target-organ damage.

Among the many circulating vasoactive factors involved in the pathophysiology of hypertension, Ang II and ET-1 are especially important.^95^^,^^96^ They are potent vasoconstrictor, mitogenic, and proinflammatory peptides that are critically involved in regulating the cardiovascular system. Activation of their respective vascular G protein–coupled receptors results in Nox activation and increased generation of ROS, which if uncontrolled leads to oxidative stress and stimulation of vascular signalling pathways such as MAPK, PKC, phospholipase C, cellular Src, and Rho kinase (Fig. 2). Increased vascular ROS bioavailability induced by Ang II, ET-1, and other vasoactive peptides also cause activation of Ca^2+^ channels, leading to accumulation of intracellular Ca^2+^ which in turn activates Ca^2+^-sensitive Nox isoforms in the vasculature, specifically Nox5, promoting a feedforward system amplifying oxidative signalling and vascular damage. Many of these systems are up-regulated in hypertension. We recently identified transient receptor potential melastatin 2 cation channel as an important Ca^2+^ channel that acts as a link between Ca^2+^ and redox signalling, a phenomenon that is increased in VSMCs in hypertension.^97^ Vascular oxidative stress is also associated with altered phosphatase activity that further amplifies kinase signalling and thus contributes to vascular damage in hypertension.^18^

In Ang II–induced hypertension in mice and rats, expression of Nox subunits, activity of Noxs, ROS generation, and oxidation of signalling molecules are increased.^18^ These processes cause oxidative damage of cells and tissues with consequent endothelial dysfunction, renal injury, and cardiovascular remodelling, processes that are attenuated by angiotensin-converting enzyme inhibitors, ROS scavengers, and Nox inhibitors.^98^ Moreover, in mice deficient in Nox2 and p47phox, the blood pressure–elevating effect of Ang II is blunted.^99^^,^^100^ These findings confirm the central role of ROS in Ang II–mediated vascular dysfunction and hypertension.

In addition to influencing Ang II vascular effects, ROS are important mediators of ET-1–induced cardiovascular dysfunction and hypertension. In mice overexpressing ET-1 in an endothelial-specific manner, blood pressure was increased in an ET type A receptor (ETAR)–dependent manner.^101^ This was associated with reduced renal artery flow, mesenteric small artery stiffening, endothelial dysfunction, vascular inflammation, and oxidative stress.^101^ In mice with pressure overload and left ventricular hypertrophy induced by transverse aortic coarctation, cardiac and coronary microvascular dysfunction were causally linked to enhanced ET-1–induced vasoconstriction, Rho kinase activation, and oxidative stress.^102^ In sunitinib-induced hypertension, we showed that endothelial dysfunction and arterial remodelling involved ET-1/ETAR–mediated Nox activation and vascular oxidative stress.^103^ Although ET-1 influences cardiovascular function through ROS generation, ROS themselves regulate the endothelin system. In Fischer 344 rats infused with a superoxide dismutase mimetic (AEOL 10150), plasma oxidative stress markers and levels of ET-1 were reduced in a dose-dependent manner.^104^ Clinically, serum ET-1 levels correlated with biomarkers of oxidative stress in patients with hypertension.^105^

### Growth factors

Many of the cardiovascular effects of Ang II and ET-1 are amplified by growth factors in hypertension. Growth factors such as EGF, IGF-1, and PDGF, which signal through their cognate receptor tyrosine kinases, induce activation of mitogenic pathways through ROS-dependent events.^106^, ^107^, ^108^, ^109^, ^110^ These processes are also stimulated by Ang II and ET-1, which transactivate growth factor receptors through various mechanisms, including A disintegrin and metalloproteinase 17 (ADAM17)–dependent shedding of growth factors, tyrosine kinase phosphorylation, and ROS107 (Fig. 2). Vascular smooth muscle cells from SHRs exhibit increased cell proliferation through Ang II/Ang II type 1 receptor (AT1R)– and ET-1/ETAR–induced transactivation of EGFR.^110^, ^111^, ^112^ These processes involve increased Nox activity, oxidative stress, activation of cellular Src, and EGFR-mediated activation of MAPKs.^111^ Ang II also transactivates the IGF-1 receptor and PDGF receptor in VSMCs through ROS generation and activation of PI3K, p38MAPK, and ERK5 pathways, leading to vascular hypertrophy and fibrosis.^107^^,^^110^^,^^113^^,^^114^ The cross-talk between G protein–coupled receptor and GFR signalling is up-regulated in hypertension and is influenced by ROS both upstream and downstream of receptor tyrosine kinase signaling.^107^ Accordingly, oxidative stress may be a common mechanism driving the amplified hypertrophic, fibrotic, and inflammatory responses induced by Ang II, ET-1, and growth factors in hypertension.^115^

## Aldosterone, Oxidative Stress, and Hypertension

An important component of the renin-angiotensin system in blood pressure regulation is Ang II stimulation of adrenal cortical cells to produce aldosterone, which signals through renal mineralocorticoid receptors (MRs) to regulate body electrolyte and fluid homeostasis.^116^ Aldosterone also signals through extrarenal MRs that influence vascular tone, adipose tissue function, cardiac contraction, and cardiovascular fibrosis.^117^^,^^118^ High levels of aldosterone are associated with hypertension, obesity, and increased risk of cardiovascular and cardiometabolic disease.^119^^,^^120^ Primary hyperaldosteronism accounts for 5%-15% of patients with hypertension.^121^ Experimental models of MR-dependent hypertension (deoxycorticosterone acetate/salt and aldosterone/salt rodents) exhibit oxidative stress as evidenced by increased Nox expression/activity, increased vascular ROS production, and elevated levels of TBARS, effects that are ameliorated by treatment with MR antagonists.^122^, ^123^, ^124^, ^125^

Aldosterone signals through its MRs through genomic and nongenomic pathways, with increasing evidence indicating a critical role for ROS in these processes. It increases ROS production in cultured VSMCs^126^^,^^127^ and endothelial cells.^128^ In VSMCs, aldosterone increases O_2_^−^ production primarily through up-regulation of Nox1,^127^^,^^129^ whereas in endothelial cells Nox4 seems to be more important.^130^ Nongenomic Nox-induced ROS generation by aldosterone/MR involves cellular Src and Rac-1, as we demonstrated in VSMCs from SHRs.^131^ In addition, there is tight interplay between Ang II and aldosterone redox signalling. Blockade of MRs inhibits Ang II–induced ROS production in vascular tissue,^122^ and AT1R is required for MR-induced endothelial dysfunction, vascular remodelling, inflammation, and oxidative stress in hypertension.^132^ In cardiomyocytes, interactions between MRs and AT1Rs participate in aldosterone-induced ROS generation through G protein–coupled receptor kinase 2–regulated Nox4.^133^

Aldosterone is a potent profibrotic hormone involved in cardiac and vascular fibrosis and remodelling in hypertension. These effects are Nox1-ROS dependent^129^ and involve nongenomic and genomic signalling through increased expression/activity of adhesion molecules (intercellular adhesion molecule 1 [ICAM-1], vascular cell adhesion molecule 1 [VCAM-1]), osteopontin, plasminogen activator inhibitor 1 (PAI-1), and growth factors. Further supporting a role for ROS in aldosterone-mediated actions are *in vitro* studies showing that MR antagonists decrease expression of Nox isoforms and subunits and attenuate oxidative stress.^134^^,^^135^ Moreover, *in vivo* studies showed that antioxidants blunt blood pressure–elevating effects of aldosterone and, in mice overexpressing MR, that hypertension is associated with oxidative stress, effects that are absent when MR is knocked out in an endothelial-specific manner.^136^ In addition, cardiomyocyte-specific overexpression of human MR induces severe coronary endothelial dysfunction with decreased NO-mediated relaxing responses to acetylcholine in coronary arteries (but not in peripheral arteries), effects prevented by MR antagonists, vitamin E/vitamin C, or a Nox inhibitor.^137^

Beyond its renal and cardiovascular effects, aldosterone influences immune cells and adipocytes, which also affect cardiovascular fibrosis and inflammation in hypertension. MRs are expressed in macrophages and T cells, where they function as a transcriptional regulator of cellular phenotype and function. The relationship between immune cells, MRs, and oxidative stress in hypertension was clearly demonstrated in aldosterone/salt–treated mice, which exhibited increased H_2_O_2_ production, up-regulation of oxidative stress–inducible tyrosine phosphatase and manganese-dependent SOD genes, and increased 3-nitrotyrosine expression in lymphocytes together with CD4+ inflammatory cells invading intramural coronary arteries.^138^ Some of the proinflammatory cardiovascular effects of aldosterone have been attributed to activation of macrophage MRs and adipocyte MRs.^139^^,^^140^ In adipocytes, aldosterone-induced MR activation causes Nox2 activation, ROS production, and activation of inflammatory pathways. In adipocytes, aldosterone/MR stimulates production of proinflammatory adipokines and ROS, which are especially important in vascular dysfunction in obesity-associated hypertension.^140^

Oxidative stress in different cell types, including VSMCs, endothelial cells, cardiomyocytes, renal cells, immune cells, and adipocytes emerges as an important player contributing to aldosterone/MR–induced cardiovascular dysfunction and damage associated with hyperaldosteronism. Accordingly, it may be possible that some of the vasoprotective and antihypertensive effects of MR antagonists, such as epleronone and spironolactone, may be mediated, at least in part, by inhibiting aldosterone/MR–induced oxidative stress.

## Oxidative Stress and Inflammation in Hypertension

The importance of inflammation in cardiovascular disease was first suggested by Ross in the 1990s, who showed that excessive inflammatory-fibroproliferative responses to various forms of insult to the endothelium and smooth muscle of the artery wall are critically involved in atherogenesis.^141^ There is now extensive experimental and clinical evidence indicating that hypertension is associated with inflammation, fibrosis, and activation of immune cells, processes that are driven in large part by oxidative stress.^142^ Tissue expression of adhesion molecules (VCAM-1, ICAM-1), production of inflammatory mediators (monocyte chemotactic peptide 1, tumour necrosis factor, interleukin [IL] 1, IL-6, 1L-17), activation of proinflammatory signalling pathways (MAPK, STAT) and transcription factors (NF-κB, AP-1, HIF-1), and circulating levels of inflammatory biomarkers (C-reactive protein, PAI-1, ILs) are increased in hypertension.^143^, ^144^, ^145^, ^146^ Although it still remains unclear whether inflammation is a cause or an effect of hypertension, it is clear that the immune system and ROS are important players.

## Immunoinflammation, Oxidative Stress, and Hypertension

The importance of the immune system in the pathophysiology of hypertension relates primarily to its effects on inflammation, which is involved in the initiation, progression, and exacerbation of cardiovascular tissue damage and remodelling.^147^ Once activated, immune cells generate high levels of ROS through Nox-dependent mechanisms, leading to cytokine production and infiltration of immune cells into the vascular wall, kidney, and heart, causing tissue damage in hypertension.^148^ The damaging effects of immune cell activation is the consequence of a shift in balance between proinflammatory and antiinflammatory cytokines and mediators and involve cells of both the adaptive (CD8+ T cells, CD4+ T cells [T_H_1, T_H_2, T_H_17, Treg cells), B cells] and innate immune systems (macrophages, monocytes, microglia, dendritic cells). As part of the innate immune system, inflammasomes seem to be especially important in inflammation, with increasing evidence suggesting a role for ROS-induced regulation of inflammasomes in hypertension^149^^,^^150^ The NLRP3 inflammasome platform may play a key role in coordinating inflammatory responses in hypertension, especially in the context of caspase-1–, IL-1β–, and IL-18–mediated reactions. Activation of NLRP3 inflammasome through redox-dependent processes has been shown in Ang II–mediated and DOCA/salt hypertension and in preeclampsia, pulmonary hypertension, and hypertension-associated kidney dysfunction.^151^, ^152^, ^153^, ^154^

The importance of the immune system and oxidative stress in hypertension has been studied in various immunodeficient mouse models. The adaptive immune system in the pathophysiology of hypertension was first demonstrated in Rag1^−/−^ mice, which lack B and T lymphocytes.^155^ In these immunodeficient mice, development of hypertension and generation of ROS induced by Ang II and DOCA/salt were blunted, effects that were reversed with adoptive transfer of T but not with B cells.^155^ Similar blood pressure responses were observed in mice deficient in macrophage colony–stimulating factor (op/op^−/−^ mice).^156^ These mice, which lack macrophages, are resistant to Ang II–induced hypertension and have reduced ROS generation and vascular inflammation compared with macrophage-intact mice. In monocyte-deficient mice, pressor effects and vascular dysfunction induced by Ang II infusion were blunted, responses that were restored with adoptive transfer of wild-type monocytes.^157^ Together, these studies, among many, clearly indicate an important role for the adaptive and innate immune systems and oxidative stress in hypertension. Multiple factors have been implicated in the activation and regulation of immune cells in hypertension, including catecholamines, Ang II, salt, ROS, and neoantigens, as discussed in detail in the current issue of this journal.

## Conclusion

There has been enormous progress in the understanding of cardiovascular, renal, and neural mechanisms involved in the pathophysiology of hypertension. Over the past decade, many new systems and factors have been identified as being important in the development of hypertension and hypertension-associated target-organ damage, including the immune system, inflammation, sex hormones, microRNAs, interstitial sodium, the microbiome, and environmental stressors. Common to these processes is oxidative stress with associated abnormal redox status and altered redox signalling. Here we have provided a unifying paradigm whereby oxidative stress acts as a common mediator of cell injury and inflammation in multiple systems that influence blood pressure regulation. Although the exact causes of oxidative stress in hypertension remain unclear, dysregulation of Noxs in cardiovascular, renal, immune, and neural cells seems to be important. The most significant consequence of oxidative stress is increased posttranslational oxidation of proteins and perturbed redox-dependent signalling. To fully understand the functional impact of oxidative stress in health and disease, it will be essential to know how proteins are differentially oxidised and activated.^158^, ^159^, ^160^ This will demand high-fidelity redox proteomics, which we believe is the next frontier in the unravelling of mechanism-specific targets in hypertension.

## Funding Sources

The authors are funded by grants from the 10.13039/501100000274British Heart Foundation (BHF; RE/18/6/34217), R.M.T. is supported through a 10.13039/501100000274BHF Chair award (CH/12/29762), and A.C.M. is supported through a Walton Foundation fellowship, 10.13039/501100000853University of Glasgow.

## Disclosures

The authors have no conflicts of interest to disclose.
